# Impact of Omega-3 and Vitamin D Supplementation on Bone Turnover Markers in Children with Leukemia: Follow-Up During and After Supplementation

**DOI:** 10.3390/nu17152526

**Published:** 2025-07-31

**Authors:** Lourdes Barbosa-Cortés, Sharon B. Morales-Montes, Michelle Maldonado-Alvarado, Jorge A. Martin-Trejo, Salvador Atilano-Miguel, Emmanuel Jiménez-Aguayo, Fabián I. Martínez-Becerril, Víctor M. Cortés-Beltrán, Atzin V. Hernández-Barbosa, Karina A. Solís-Labastida, Jorge Maldonado-Hernández, Benito A. Bautista-Martínez, Azalia Juárez-Moya, Zayra Hernández-Piñón, Juan M. Domínguez-Salgado, Judith Villa-Morales, Israel Domínguez-Calderón

**Affiliations:** 1 Unidad de Investigación Médica en Nutrición, Unidad Médica de Alta Especialidad (UMAE), Instituto Mexicano del Seguro Social (IMSS), Hospital de Pediatría, Centro Médico Nacional Siglo XXI, Ciudad de México 06720, Mexico; sharon03102001@gmail.com (S.B.M.-M.); andymich1530@gmail.com (M.M.-A.); atilanosalvador@gmail.com (S.A.-M.); emmanueljimenezag@gmail.com (E.J.-A.); fabian-37@hotmial.com (F.I.M.-B.); victorcortesbeltran@gmail.com (V.M.C.-B.); vicky_96_draco@yahoo.com.mx (A.V.H.-B.); jormh@yahoo.com.mx (J.M.-H.); jmdomin6@outlook.com (J.M.D.-S.); jusyvm@hotmail.com (J.V.-M.); israfquimica21@gmail.com (I.D.-C.); 2 Sección de Estudios de Posgrado, Escuela Superior de Medicina, Instituto Politécnico Nacional, Ciudad de México 11340, Mexico; 3 Departamento Clínico de Hematología, Unidad Médica de Alta Especialidad (UMAE), Instituto Mexicano del Seguro Social (IMSS), Hospital de Pediatría, Centro Médico Nacional Siglo XXI, Ciudad de México 06720, Mexico; jorge.martin.trejo@gmail.com (J.A.M.-T.); kas_anastacia@yahoo.com (K.A.S.-L.); bbautistamartinez@yahoo.com (B.A.B.-M.); dra.ajuarez@gmail.com (A.J.-M.); zayra_hp@hotmail.com (Z.H.-P.); 4 Facultad de Ciencias de la Salud, Universidad Anáhuac, Ciudad de México 52786, Mexico

**Keywords:** acute lymphoblastic leukemia, children, *n*-3 polyunsaturated fatty acids, vitamin D, bone turnover markers, dietary supplements

## Abstract

**Background/Objective**: In patients with acute lymphoblastic leukemia (ALL), it has been demonstrated that the treatment has a negative effect on bone health. The *n*-3 polyunsaturated fatty acids (LCPUFAs-ω3) may attenuate bone resorption. We evaluated the effects of LCPUFAs-ω3, vitamin D, and calcium supplementation on bone turnover markers and changes in vitamin D concentrations during 6 weeks of supplementation and during 6 weeks of post-intervention follow-up in pediatric patients with ALL. **Methods**: Thirty-six pediatric patients with ALL were randomly assigned to the ω-3VDCa group (100 mg/kg/d LCPUFAs-ω3 + 4000 IU vitamin D + 1000 mg calcium) or the VDCa group (4000 IU vitamin D + 1000 mg calcium) for 6 weeks. Blood samples were collected to determine 25(OH)D, PTH, ICTP, and TRAP-5b (biomarkers of bone resorption) and osteocalcin (OC, a biomarker of bone production) levels at baseline, 6 weeks, and 12 weeks after supplementation. The 25(OH)D analysis was performed using ultra-high-performance liquid chromatography coupled to a mass spectrometer, and PTH and bone turnover markers were measured by ELISA. **Results**: The 25(OH)D concentration increased in both groups (ω3VDCa group: 19.4 ng/mL vs. 44.0 ng/mL, *p* < 0.0001; VDCa group: 15.3 ng/mL vs. 42.8 ng/mL, *p* = 0.018) and remained significantly higher at 12 weeks. At 12 weeks, ICTP showed lower concentrations in the ω-3VDCa group than in the VDCa group (0.74 ng/mL vs. 1.05 ng/mL, *p* = 0.024). **Conclusions**: Combined omega-3 and 4000 IU vitamin D supplementation for 6 weeks had a positive effect on bone health, as indicated by serum ICTP, with no effect on serum 25(OH)D levels over vitamin D supplementation alone.

## 1. Introduction

The survival rate of children and adolescents with acute lymphoblastic leukemia (ALL) has progressively improved in recent decades [1]. However, this has led to the development of many comorbidities in ALL survivors. Glucocorticoids are frequently prescribed as some of the most powerful osteotoxic drugs in pediatric patients with ALL; however, they have been associated with a negative impact on bone health [1] and generate bone morbidities such as osteoporosis. Mesenchymal stem cells (MSCs) such as osteoblasts, osteocytes, and osteoclasts are impacted by these drugs, due to their multiple mechanisms of action underlying the changes in several remodeling processes, including PPARγR2 upregulation, increased sclerostin expression, an increased Receptor Activator of Nuclear Factor κB Ligand and osteoprotegerin ratio (RANKL/OPG), and altered renal and intestinal calcium handling [1,2].

Childhood and adolescence are important periods in the attainment of peak bone mass, which can be impaired by various factors, such as nutritional status, drug use, and the presence of neoplasms, increasing the risk of developing osteoporosis and fractures in adulthood [3].

In addition, low bone mineral density and fractures have been partly attributed to vitamin D deficiency during treatment for pediatric ALL. Due to long inpatient stays, decreased outdoor activities (reducing exposure to sunlight and impairing vitamin D synthesis), and a lack of appetite, vitamin D intake is decreased, leading to this deficiency [4]. The maintenance of calcium and phosphorus homeostasis and bone health in childhood and adolescence is greatly impacted by vitamin D [5] because it has direct effects on the main cells involved in bone metabolism, stimulating osteoblast differentiation and the synthesis of proteins involved in calcium deposition, thus increasing bone matrix mineralization [6].

Previous studies have reported that vitamin D levels decrease in children with leukemia undergoing treatment [7,8]. Our group of collaborators recently reported a high frequency of vitamin D deficiency > 90% in the early stages of treatment, in addition to an increase in bone resorption markers, showing that these patients experience alterations in bone remodeling [5]. For this reason, pediatric patients with ALL are candidates for nutritional interventions that reverse bone deterioration, such as vitamin D and calcium supplementation.

However, the ideal dose, time of supplementation, and optimal levels of vitamin D remain controversial [9,10,11,12]. Previous studies have shown discrepancies in the impact of vitamin D supplementation on bone mineral density, bone formation, and resorption markers.

Different studies have demonstrated that omega-3 polyunsaturated fatty acids (LCPUFAs-ω3), such as docosahexaenoic acid (DHA) and eicosapentaenoic acid (EPA), have great benefits for treating and preventing various diseases, including cardiovascular, neurodegenerative, cancer, and bone diseases. More recently, it has been suggested that they help to decrease bone loss and the risk of osteoporosis by inhibiting the production of inflammatory cytokines, such as IL-1, IL-6, and TNF-α. Furthermore, LCPUFAs-ω3 have been reported to inhibit osteoclast activity, which has been shown to enhance bone health. Macrophage colony-stimulating factor (MCSF), microphthalmia-associated transcription factor (MITF), and Receptor Activator of Nuclear kB (RANK) expression can be inhibited by fish oil administration, inhibiting bone resorption. Another mechanism by which LCPUFAs-ω3 attenuate osteoclast activity is by decreasing the production of prostaglandin E2; this decrease has been shown to favor increased osteoblastogenesis [13].

In pediatric patients with ALL, LCPUFAs-ω3 supplementation has been reported to have positive effects, for example, lowering lipid levels, maintaining body composition, and decreasing cardiovascular risk [14,15,16,17]. In this sense, it has been suggested that LCPUFAs-ω3 play an important role in bone metabolism and may thus represent a non-pharmacological (nutraceutical) means of reducing alterations in bone metabolism. To the best of our knowledge, information about nutritional interventions with vitamin D in pediatric patients with ALL has been limited to reports describing only the changes that occur during the supplementation period, with no reports describing how long the effect of supplementation with LCPUFAs-ω3, vitamin D, and calcium on vitamin D nutritional status and bone turnover markers lasts after supplementation ends. Therefore, we evaluated the effect of daily LCPUFAs-ω3 supplementation combined with vitamin D and calcium on bone turnover markers and changes in vitamin D concentrations during 6 weeks of supplementation followed by 6 weeks of post-intervention follow-up in pediatric patients with acute lymphoblastic leukemia.

## 2. Materials and Methods

### 2.1. Study Design 

This randomized controlled clinical trial was conducted in compliance with the guidelines established by the Declaration of Helsinki [18] and was approved by the Research and Ethics Committee of the Instituto Mexicano del Seguro Social (IMSS) in Mexico City (approval # 2019-785-021). This trial is registered on the clinicaltrials.gov platform (NCT05950204). Data collection began after all the parents or legal guardians of the children provided written informed consent.

### 2.2. Patients

One hundred and eight patients with ALL in the maintenance phase were eligible for the study, which was conducted at the Pediatric Hospital, Centro Médico Nacional SXXI, Instituto Mexicano del Seguro Social (IMSS), in Mexico City, from September 2022 to August 2024. The exclusion criteria were as follows: children who had fish allergies or those who were unable to swallow capsules (LCPUFAs-ω3); children with Down syndrome or hypersensitivity to cholecalciferol or metabolites of vitamin D3; children with hyperphosphatemia, hypercalcemia, hypercalciuria, or calcium lithiasis; and children who routinely consumed vitamin supplements or LCPUFAs-ω3. A total of 94 patients were excluded because they did not meet the inclusion criteria, and 14 were excluded because they refused to participate. Therefore, 36 patients (5.0–17 years) were randomized. Patients were classified into 3 groups according to the following characteristics: SR included good steroid responses, ages > 1 year and <7 years, initial white blood cell counts less than 20,000/mm^3^, bone marrow in remission at day +33, no immunophenotype T, and no HR criteria; IR comprised ages > 7 to <10 years, initial white blood cell counts of 20,000/mm^3^ to <50,000/mm^3^, immunophenotype T, no HR criteria, HR patients with poor response to prednisone, initial white blood cell counts >50,000/mm^3^, and no response to induction on day +33, t(9;22), and t(4;11).

At our institute, we use the HP09 chemotherapy protocol, based on BFM95. The patients began treatment with 50 mg/m^2^ of prednisone monotherapy daily for 7 days. Then, they began the remission induction phase (lasting for 29–33 days), during which they received 60 mg/m^2^ of prednisone, 1.5 mg/m^2^ of vincristine, 30 mg/m^2^ of daunorubicin, 5000 IU/m^2^ of L-asparaginase, and intrathecal chemotherapy. During the consolidation phase, they received 60 mg/m^2^ of 6-mercaptopurine, 1000 mg/m^2^ of cyclophosphamide IV, 75 mg/m^2^ of ARA-C IV, and intrathecal chemotherapy. Additionally, high-risk patients received 20 mg/m^2^ of dexamethasone and 25,000 IU/m^2^ of L-asparaginase. The intensification phase included 60 mg/m^2^ of prednisone, 1.5 mg/m^2^ of vincristine, 30 mg/m^2^ of daunorubicin, and 5000 IU/m^2^ of L-asparaginase. Finally, in the maintenance phase, all patients received 50 mg/m^2^ of 6-mercaptopurine and 20 mg/m^2^ of oral methotrexate [19].

### 2.3. Sample Size

The sample size was calculated for the outcomes of bone metabolism markers, such as 25-hydroxyvitamin D (25(OH)D), osteocalcin (OC), and human cross-linked *C*-terminal telopeptides of type I collagen (ICTP). The sample size was calculated based on the mean and standard deviation, and a z-alpha value of 0.05 and a beta value of 0.80 were considered, using the formula for the mean difference. Considering the potential of 20% attrition, the final sample size was 18 subjects per group.

### 2.4. Recruitment and Allocation

The selected children were recruited, screened, and assigned (1:1) to either the VDCa group or the ω-3VDCa group using a computer-generated random number list with software for parallel groups (Random Allocation Software) [20]. The randomization process utilized balanced blocks with ten children in each group. During the study, physicians, researchers, and nutritionists remained unaware of the treatment allocations. A technician without blinders managed the randomization process. Investigators were blinded to group allocation until the study was concluded.

### 2.5. Intervention

LCPUFAs-ω3 (EPA + DHA) were administered at a rate of 100 mg/kg/d capsules as natural triglyceride soft gels made of gelatin, without any artificial color or flavor, which were molecularly distilled, with a maximum dose of 3 g/d containing 225 mg of DHA, 325 mg of EPA, and 90 mg of other LCPUFAs-ω3 per capsule (Nordic Naturals, Inc., Watsonville, CA, USA). The capsules were swallowed with water. The LCPUFAs-ω3 complied with the principles established for fats according to the European Pharmacopoeia Standard (EPS) and according to the Council for Responsible Nutrition (CRN) and the Global Organization (CRNGO), in which they are considered safe products that do not exceed the maximum allowances for contaminants such as peroxides, heavy metals, dioxins, and PCBs. All the capsules contained 30 mg of vitamin E as an antioxidant. Calcium was administered as calcium carbonate (CALCID^®^) at 1000 mg/day orally in cherry, orange, or lemon flavor and chewed. Vitamin D was administered orally as cholecalciferol at 4000 IU (100 µg/day) (Histofil^®^), dissolved in water in advance of offering it to the child. The VDCa group received the same doses of vitamin D and calcium. The supplementation duration was 6 weeks, and all the children were re-evaluated 6 weeks after the intervention. To maintain adequate blood levels with minimal sunlight exposure, for children aged 1–18 years who are vitamin D-deficient, supplementation with 2000 IU/d of vitamin D for at least 6 weeks has been suggested. However, children receiving glucocorticoid treatment could be given at least two to three times more vitamin D for their age group to satisfy their body’s vitamin D requirement. According to the Endocrine Society Guidelines, the maintenance tolerable upper limits of vitamin D, 4000 IU/d for children 1–18 years, may be needed to correct vitamin D deficiency [21,22,23,24].

During the study period, supplementation was monitored by phone or in the hospital if necessary for medical reasons. The side effects presented by the children (constipation, vomiting, nausea, diarrhea, burps, or headache) during the intervention were documented and registered by one of the researchers.

### 2.6. Supplementation Compliance

At the start of the intervention, children and their parents were asked to track their supplement intake (capsules and pills) in a logbook. Compliance was assessed by checking the leftover pills and capsules during the following appointment. Only those children who consumed at least 80% of the LCPUFAs-ω3 capsules were considered for inclusion. Furthermore, the levels of EPA and DHA in erythrocytes were measured before and 6 and 12 weeks after supplementation began to ensure adherence to the treatment.

Vitamin D adherence was assessed according to changes in the vitamin D nutritional status after supplementation.

### 2.7. Procedures

#### 2.7.1. Anthropometry

The clinical and demographic characteristics of the enrolled patients were recorded during the baseline measurement (at maintenance) and at 6 and 12 weeks of follow-up. InBody 230 equipment (InBody USA, Cerritos, CA, USA) was used to determine body weight and body composition. Height was determined with a Seca 222 wall-mounted stadiometer (Seca Corp., Oakland Center, Columbia, MD, USA). Body mass index (BMI) was obtained by dividing weight (kg) by the square of height (m), which was classified according to World Health Organization (WHO) guidelines [25]. These measurements were carried out by trained personnel according to standard techniques at the 3 measurement times of the study.

#### 2.7.2. Analytical Methods

Peripheral blood samples were obtained at baseline and 6 and 12 weeks with an 8 h fast. The samples were centrifuged for 15 min at 3500 rpm at 4 °C. The serum and plasma obtained were stored at −80°C until analysis. The serum 25(OH)D concentration was determined using ultra-high-performance liquid chromatography coupled to a mass spectrometer (UPLC-MS-MS). The UPLC-MS-MS equipment consisted of an ACQUITY UPLC Class H system with a photodiode array detector (PDA) and a mass spectrometer (ACQUITY QD) (Waters, Milford, MA, USA) in an electrospray ionization (ESI) mode, in addition to a quaternary eluent management system. The vitamin D nutritional status was established according to the Endocrine Society cut-off points, considering deficiency to be <20 ng/mL, insufficiency to be 21–29 ng/mL, and sufficiency to be ≥30 ng/mL [23]. The parathyroid hormone (PTH) and OC concentrations were determined using a MILLIPLEX^®^ Human Bone Magnetic Bead Panel (HBNMAG-51K) (Merck KGaA, Darmstadt, Germany), with an analytical sensitivity of 1.8 pg/mL and a detection range of 5 pg/mL–20,000 pg/mL for PTH and an analytical sensitivity of 68.5 pg/mL and a detection range of 146 pg/mL–600,000 pg/mL for OC. Human tartrate-resistant acid phosphatase 5b (TRAP-5b) was assessed using a commercial kit (MBS045195; MyBiosource Inc., San Diego, CA, USA), with an analytical sensitivity of 0.1 U/L and a detection range of 0.5 U/L–16 U/L. Human cross-linked *C*-terminal telopeptides of type I collagen (ICTPs) were assessed in the plasma using a commercial kit (MBS040005; MyBiosource Inc., San Diego, CA, USA), with an analytical sensitivity of 0.1 ng/mL and a detection range of 0.625 ng/mL–20 ng/mL.

Serum calcium (REF. 1001060, reference values in children were 10 mg/dL–12 mg/dL), phosphorus (REF. 1001156, reference values in children were 4.0 mg/dL–7.0 mg/dL), and creatinine were measured. The calcium in urinary samples was determined by spectrophotometry (SPINREACT 120, Santa Coloma, España). Urinary samples were also obtained for calciuria measurements, which were estimated in isolated urine by determining the calcium/creatinine (Ca/Cr) ratio, expressed in mg/mg or mmol/mmol. In children older than two years, a ratio higher than 0.2 mg/mg or 0.6 mmol/mmol suggests hypercalciuria.

#### 2.7.3. Fatty Acid Analyses Using Gas Chromatography

Analyses were performed with a 7820A gas chromatograph (Agilent Technologies, Santa Clara, CA, USA) with a flame ionization detector (FID), as described previously [14].

#### 2.7.4. Evaluation of Bone Mineral Density

The bone mineral density (BMD) in the lumbar spine vertebrae and total body was measured by dual-energy X-ray absorptiometry (DXA) using a GE Lunar Prodigy Advance scanner (software version 9.0; GE Medical Systems, Madison, WI, USA) at baseline only due to the short intervention duration. The parameters included the total bone mineral content (BMD) (g/m^2^) and the z-score of the BMD at the lumbar spine level and total body. The BMD z-score was adjusted for height and sex. The BMD was considered normal for z-scores > −1 SD, indicative of osteopenia between –1 SD and −2 SD, and indicative of osteoporosis ≤ −2 SD [10].

#### 2.7.5. Statistical Analysis

SPSS Statistics version 21.0 software was used to perform a statistical analysis (SPSS Inc., Chicago, IL, USA). Asymmetric coefficients of −0.5 and +0.5 and kurtosis coefficients between −2 and +2 were evaluated by the Shapiro–Wilk test for the data distribution. The quantitative data are presented as mean standard deviations (SDs) or medians (25th–75th percentiles) according to normality tests. Categorical variables are presented as percentages. To analyze the changes in 25(OH)D, OC, TRACP-5B, ICTP, PTH, calcium, and phosphorus levels during the follow-up, we used the Wilcoxon test or the paired Student *t* test according to the data distribution. The differences between groups were evaluated using Student’s *t* test or the Mann–Whitney U test. The association between changes in 25(OH)D concentrations, LCPUFAs-ω3 enrichment, and bone turnover marker concentrations was analyzed using a multiple linear regression model.

## 3. Results

**Figure 1:**
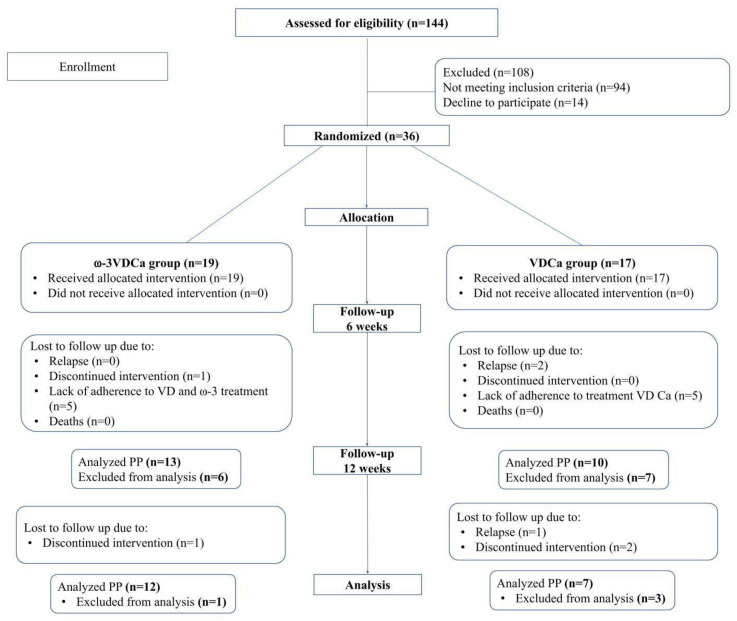
Flow diagram of Consolidated Standards of Reporting Trials (CONSORT) for studying patients with acute lymphoblastic leukemia. ω-3: long-chain omega-3 polyunsaturated fatty acids; VD: vitamin D; Ca: calcium; PP: per protocol.

This study was in accordance with the Consolidated Standards of Reporting Trials (CONSORT) guidelines. Of 144 eligible patients, 36 children were allowed to participate in the study and were randomly assigned to a treatment group (19 were included in the ω-3VDCa intervention group, and 17 were included in the VDCa intervention group). The causes for dropout are indicated and displayed in the same figure. Loss to follow-up was due to clinical complications such as relapses, discontinued intervention, or a lack of adherence (Figure 1).

### 3.1. Demographic and Clinical Characteristics of All Randomized and Study-Completing Children by Protocol

The demographic and clinical characteristics of all the randomized and study-completing children at baseline and at 6 and 12 weeks are shown. The median age of the children at baseline was 9 and 10 years in the ω-3VDCa and VDCa groups, respectively. During the follow-up, most of the children were classified as eutrophic and at a high risk of relapse (Table 1).

As a control marker for supplementation, we used the calcium/creatinine ratio, which increased in the 6 weeks after supplementation (0.01 mg/dL vs. 0.06 mg/dL, *p* = 0.014) and significantly decreased at 12 weeks without supplementation (0.04 mg/dL, *p* = 0.021) in the ω-3VDCa group. However, although the concentrations increased, they were always within an adequate range, without any patient developing hypercalciuria.

### 3.2. Compliance with Supplementation

#### 3.2.1. Vitamin D

At the study baseline, there were no significant differences in the nutritional status or 25(OH)D concentrations.

**Figure 2:**
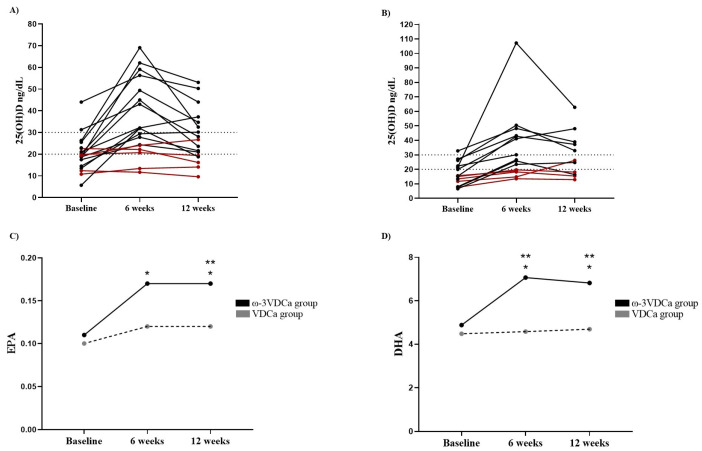
Changes in 25(OH)D concentrations and LCPUFA-ω3 incorporation into erythrocytes between baseline, 6 weeks, and 12 weeks. (**A**) Changes in 25(OH)D concentrations of the ω-3VDCa group; (**B**) changes in 25(OH)D concentrations of the VDCa group; (**C**) changes in the incorporation of EPA into erythrocytes; and (**D**) changes in the incorporation of DHA into erythrocytes. EPA: eicosapentaenoic acid; DHA: docosahexaenoic acid; ω-3VDCa group: omega-3 long-chain polyunsaturated fatty acids, vitamin D, and calcium; VDCa group: vitamin D and calcium. * EPA ω-3VDCa group, baseline vs. 6 weeks *p* = 0.001. * EPA ω-3VDCa group, baseline vs. 12 weeks *p* < 0.0001. ** EPA ω-3VDCa group 12 weeks vs. VDCa group 12 weeks *p* = 0.039. * DHA ω-3VDCa group, baseline vs. 6 weeks *p* < 0.0001. * DHA ω-3VDCa group, baseline vs. 12 weeks *p* < 0.0001. ** DHA ω-3VDCa group 6 weeks vs. VDCa group 6 weeks *p* < 0.0001. ** DHA ω-3VDCa group 12 weeks vs. VDCa group 12 weeks *p* < 0.0001. Red color: patients that did not change 25(OH)D nutritional status and black color: patients that changed 25(OH)D nutritional status.

An analysis of the total sample showed that, in 10 (ω-3VDCa group: n = 5; VDCa group: n = 5) children, no changes in 25(OH)D concentrations were observed between the baseline and 6 weeks of supplementation (the ω-3VDCa group: 19.9 ng/mL at baseline vs. 20.7 ng/mL at 6 weeks; the VDCa group: 15.6 ng/mL at baseline vs. 18.8 ng/mL at 6 weeks) (Figure 2A,B). These values show that the patients did not adhere to the vitamin D supplementation; therefore, they were removed from the analysis, as indicated in the CONSORT guidelines (Figure 1).

Based on the changes in the vitamin D nutritional status and EPA and DHA enrichment in erythrocytes, we found that 63.2% of the ω-3VDCa group presented good adherence to supplementation, while only 41.2% of the VDCa group presented good adherence. According to the capsule count of the total sample, the vitamin D adherence rate was 95.3%, with the calcium adherence rate being 93.6% and the LCPUFAs-ω3 adherence rate being 90.1%.

#### 3.2.2. Omega-3 Long-Chain Polyunsaturated Fatty Acids

At the beginning of the study, both groups had the same EPA and DHA levels (*p* = 0.271 and *p* = 0.352, respectively). After 6 weeks of supplementation, we observed a significant increase in EPA (*p* = 0.001) and DHA (*p* < 0.0001) in the ω-3VDCa group; however, there were no significant changes in the EPA and DHA concentrations in the VDCa group. In the intergroup analysis, we found that, at 6 weeks, the EPA concentrations showed a trend of becoming higher in the ω-3VDCa group than in the VDCa group (*p* = 0.070), and the DHA concentrations in the ω-3VDCa group were significantly increased (*p* < 0.0001) (Figure 2C,D).

#### 3.2.3. Omega-3 Long-Chain Polyunsaturated Fatty Acids After Supplementation (12 Weeks)

At 12 weeks after supplementation, the EPA and DHA concentrations were maintained and remained higher than baseline levels in the ω-3VDCa group. An intergroup analysis at 12 weeks revealed that the EPA and DHA concentrations were higher in the ω-3VDCa group than in the VDCa group (EPA *p* = 0.039; DHA *p* < 0.0001) (Figure 2C,D).

### 3.3. Follow-Up of Changes in Vitamin D Status During and After Supplementation

**Figure 3:**
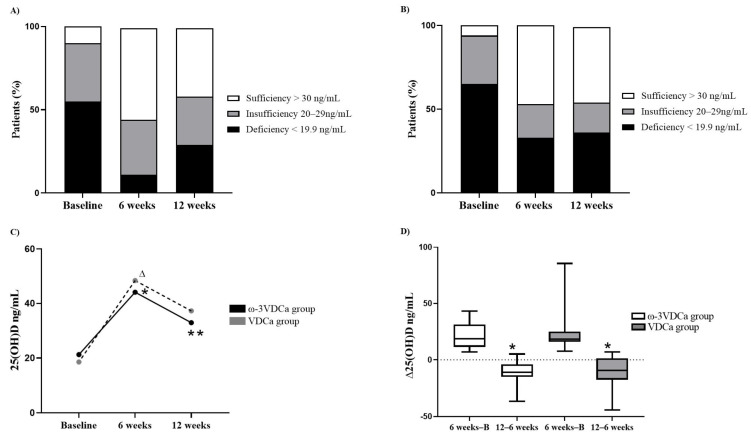
Changes in vitamin D nutritional status and concentrations at baseline, 6 weeks, and 12 weeks. (**A**) Changes in vitamin D nutritional status of the ω-3VDCa group; (**B**) changes in vitamin D nutritional status of the VDCa group; (**C**) changes in 25(OH)D concentrations; and (**D**) ∆ of 25(OH)D. ω-3VDCa group: omega-3 long-chain polyunsaturated fatty acids, vitamin D, and calcium; VDCa group: vitamin D and calcium. Data was analyzed with Student’s *t* test for related samples or the Mann–Whitney U test. Here, * 25(OH)D, ω-3VDCa group, baseline vs. 6 weeks *p* < 0.0001, and ** 25(OH)D, ω-3VDCa group, 6 weeks vs. 12 weeks. ∆ 25(OH)D, VDCa group, 6 weeks vs. baseline *p* = 0.018. * ω-3VDCa group, Δ6 weeks–baseline vs. Δ12–6 weeks *p* = < 0.0001. * VDCa group, Δ6 weeks–baseline vs. Δ12–6 weeks *p* = 0.018.

Figure 3A,B show the percentage change in the vitamin D nutritional status of the total sample. At the beginning of the study, 55% of the patients in the ω-3VDCa group presented deficient levels, and 35% presented insufficient levels. After 6 weeks of supplementation, 11% of the patients remained deficient, 33% remained insufficient, and 55% reached adequate levels (Figure 3A). At the start of the study, 65% of the children in the VDCa group had deficient levels, and 29% had insufficient levels. After 6 weeks of supplementation, 47% achieved sufficiency, 20% remained insufficient, and 33% continued to have a deficiency (Figure 3B). At the end of the 12-week follow-up without supplementation, 58.3% of the patients in the ω-3VDCa group maintained sufficient vitamin D concentrations. During the follow-up without supplementation, 71.4% of the patients in the VDCa group maintained sufficient vitamin D concentrations (Figure 3A,B).

In the per-protocol analysis, we observed a significant increase in 25(OH)D concentrations in both groups at 6 weeks (ω-3VDCa group: 19.4 ng/mL vs. 44.0 ng/mL, *p* < 0.0001; VDCa group: 15.3 ng/mL vs. 42.8 ng/mL, *p* = 0.018). There were no differences between groups at 6 weeks (*p* = 0.735). In the analysis of the ω-3VDCa group between 6 and 12 weeks, there was a decrease in 25(OH)D concentrations (44.0 ng/mL vs. 31.4 ng/mL, *p* = 0.005). However, in the VDCa group during the follow-up, no changes were found in 25(OH)D concentrations (42.80 ng/mL vs. 37.20 ng/mL, *p* = 0.091) (Figure 3C).

We observed that the 25(OH)D concentrations remained significantly higher at 12 weeks than at baseline in both groups (ω-3VDCa group: 32.9 ng/mL vs. 21.2 ng/mL, *p* = 0.008; VDCa group: 37.2 ng/mL vs. 18.5 ng/mL, *p* = 0.018) (Figure 3C).

An intragroup analysis of 25(OH)D deltas showed a significant decrease in the ω-3VDCa group (Δ6 weeks–baseline of 22.3 ng/mL vs. Δ12–6 weeks of −11.0 ng/mL, *p* = < 0.0001) and in the VDCa group (Δ6 weeks–baseline of 21.2 ng/mL vs. Δ12–6 weeks of −9.3 ng/mL, *p* = 0.018). An intergroup 25(OH)D delta analysis showed no changes (Figure 3D).

### 3.4. BMD and Vitamin D Nutritional Status

Bone mineral density was measured only at baseline in order to determine the relationship between vitamin D nutritional status and bone health at maintenance. According to the z-score of L1-L4, we found that 21 patients (58%) had an adequate BMD, 11 patients (31%) were in a range that could suggest osteopenia, and 4 patients (11%) had a low BMD that could suggest osteoporosis. Only three patients had vitamin D sufficiency at baseline, but none presented an adequate BMD (Table 2).

### 3.5. Follow-Up of Changes in PTH Status During and After Supplementation

**Figure 4:**
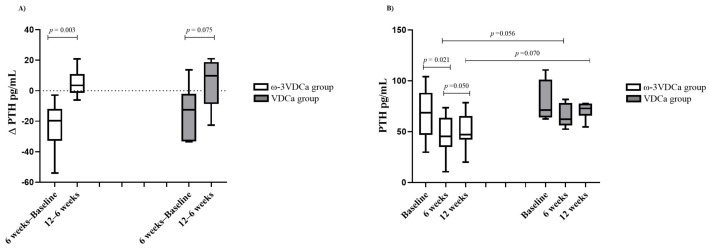
Changes in PTH concentrations at baseline, 6 weeks, and 12 weeks. (**A**) Changes in PTH concentrations; (**B**) ∆ of PTH. ω-3VDCa group: omega-3 long-chain polyunsaturated fatty acids, vitamin D, and calcium; VDCa group: vitamin D and calcium; PTH: parathormone. Analysis performed using the Mann–Whitney U test and Wilcoxon signed-rank test.

Regarding PTH, we observed that, between the baseline and 6 weeks of supplementation, there was a significant decrease in the ω-3VDCa group (77.4 pg/mL vs. 48.5 pg/mL, *p* = 0.009), and there were no changes in the VDCa group. An intragroup analysis of PTH deltas showed an increase in the ω-3VDCa group (Δ6 weeks–baseline −19.2 pg/mL vs. Δ12–6 weeks 4.9 pg/mL, *p* = 0.003) and an increasing trend in the VDCa group (Δ6 weeks–baseline −12.5 pg/mL vs. Δ12–6 weeks 9.7 pg/mL, *p* = 0.075) (Figure 4).

### 3.6. Bone Turnover Markers

**Figure 5:**
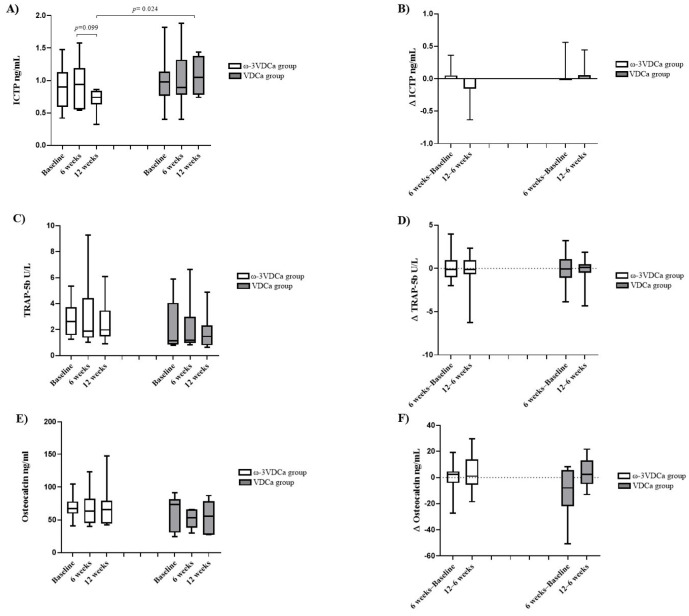
Changes in concentrations and deltas of bone turnover markers at baseline, 6 weeks, and 12 weeks. (**A**) Changes in ICTP concentrations; (**B**) ∆ ICTP; (**C**) changes in TRAP-5b concentrations; (**D**) ∆ TRAP-5b; (**E**) changes in osteocalcin concentrations; (**F**) ∆ osteocalcin. ICTP: *C*-terminal telopeptide of type I collagen.; TRAP-5b: tartrate-resistant acid phosphatase 5b; ω-3VDCa group: omega-3 long-chain polyunsaturated fatty acids, vitamin D, and calcium; VDCa group: vitamin D and calcium. Analysis performed using the Mann–Whitney U test and Wilcoxon signed-rank test.

Regarding bone turnover markers, only ICTP at 12 weeks showed lower concentrations in the ω-3VDCa group than in the VDCa group (0.74 ng/mL vs. 1.05 ng/mL, *p* = 0.024). The other bone turnover markers, such as OC and TRAP-5b, showed no changes (Figure 5). A multiple linear regression analysis at 12 weeks showed that, for each increase in the DHA percentage, there was a decrease of −0.630 ng/mL in ICTP (Table 3).

### 3.7. Diet

As part of patient follow-up, we evaluated the recommended daily intake of vitamin D, LCPUFAs-ω3, and calcium. In the analysis of the total sample, we observed that the median dietary intake of vitamin D was 164.8 UI at baseline and 176.5 UI at 6 weeks; that of LCPUFAs-ω3 was 1.2 g at baseline and 1.2 g at 6 weeks; and that of calcium was 774.8 mg at baseline and 762.0 mg at 6 weeks. In this context, we did not observe any differences in the intake of these micronutrients during the supplementation period.

## 4. Discussion

This is the first report demonstrating the effect of combined supplementation with LCPUFAs-ω3, vitamin D, and calcium for six weeks and after the intervention on 25(OH)D concentrations and bone turnover markers in pediatric patients with ALL. We confirmed a high prevalence of vitamin D insufficiency and deficiency in this population, which persisted into the maintenance phase.

The administered dose of vitamin D was effective in increasing 25(OH)D concentrations at 6 weeks in both groups. We found that, in patients who had adequate adherence, more than 50% maintained sufficient vitamin D levels 12 weeks after receiving supplementation. LCPUFAs-ω3 did not appear to have an effect on 25(OH)D concentrations after supplementation. Similarly, during supplementation with vitamin D and LCPUFAs-ω3, no effect on bone formation and resorption markers was observed; however, the ω-3VDCa group showed a significant decrease in PTH concentrations after 6 weeks of supplementation and a trend of decreasing at 6 weeks after its discontinuation. In addition, in the ω-3VDCa group, the ICTP concentrations decreased six weeks after discontinuing supplementation. In contrast, there were no differences between the groups in terms of the bone formation marker OC.

According to ENSANUT 2022, 4.3% and 23.3% of healthy Mexican preschoolers and schoolchildren have vitamin D deficiency [26]; however, various studies have found that the prevalence of vitamin D deficiency is higher in pediatric patients with ALL than in healthy children [5,8]. The high prevalence of vitamin D deficiency in this population has led to a search for therapeutic strategies, as it is necessary to maintain adequate 25(OH)D concentrations in order to regulate calcium homeostasis and maintain bone health [23,26]. To date, few studies have evaluated the effect of supplementation on bone health in these patients [9,10,11,12]. Interventions have focused mainly on administering vitamin D and calcium, and only one study combined vitamin D and K [12]. These studies reported a significant increase in 25(OH)D concentration after supplementation [10], with an adequate tolerance of the intervention [10,11], thus demonstrating the effectiveness of vitamin D supplementation in improving 25(OH)D levels and the feasibility of integrating it into hematologic treatment [11]. Even though all supplementation studies in these patients have focused on the remission induction phase to the intensification phase, it has been suggested that the effect of supplementation should be evaluated during the maintenance phase [11]. However, at this treatment stage, poor adherence to chemotherapy has been observed, representing a challenge to achieving adequate adherence to supplementation [27].

When evaluating the adherence of the total sample by counting the calcium, vitamin D, and LCPUFAs-ω3 capsules, it was found that the patients apparently complied, with an adherence rate of 90–95%; however, when evaluating adherence based on the change in concentrations from baseline, the adherence percentage was lower (53%), which suggests that determining the changes in the concentrations of these analytes is a more reliable method for evaluating adherence. It is difficult to ensure the consumption of supplements because, in the case of pediatric patients, the parents are responsible for their intake, or, in the case of adolescents, they are responsible themselves. Additionally, as these children receive polypharmacy, it is difficult for them to adhere to the intervention due to the prolonged time of their own treatment and “pill fatigue”. Some authors have suggested strategies to improve adherence to supplementation, such as using chewable calcium tablets [11], which we implemented in our study to ensure better adherence. Although we used this strategy, one patient in particular presented an increase of 12 ng/mL in 25(OH)D after supplementation had already been suspended, increasing from 14.8 ng/mL at 6 weeks to 26.2 ng/mL at 12 weeks, which could suggest that the supplements were consumed after the supplementation period ended. Some studies have reported that, for every 100 IU of vitamin D consumed, there is a replacement of 0.75 ng/mL, and it has been suggested that taking a supplement with more than 1000 IU could ensure sufficient levels [28]. It should be noted that the vitamin D and calcium intake in these patients was ~ 40% and 70%, respectively, which indicates that none of the patients met the recommended requirements of 400 IU of vitamin D and 1000 mg of calcium. On the other hand, free participation, surveillance, strict control of their health status, and the opportunity to correct specific nutrient deficiencies, as in our case, which are not performed in the routine clinic, are not factors that guarantee adherence. In our case, the environmental setting plays a determining role in adherence to treatment in pediatric leukemia patients.

Previous studies administering vitamin D supplementation have demonstrated its effectiveness in increasing 25(OH)D concentrations at doses between 400 and 800 IU [9,10] or at a high dose of 100,000 IU [11]. Although vitamin D supplementation has been recommended to prevent osteoporosis and reduce the risk of fractures, the required dose, how it should be administered, and the optimal levels of 25(OH)D required to maintain adequate bone health remain controversial [6,29]. Maintaining serum vitamin D levels of 40–60 ng/mL has been described as providing health benefits; to achieve these serum levels, supplementation at doses of 4000 IU per day has been suggested in subjects at risk of vitamin D deficiency [29,30]. Our results showed a significant increase in 25(OH)D concentrations, with sufficient concentrations reached and even maintained 6 weeks after supplementation stopped. Although a decrease in 25(OH)D concentrations was observed when supplementation was stopped in both groups, they were still greater than those at baseline, with sufficient concentrations maintained in more than 50% of patients. In contrast, in a study by Demirsoy et al., 400–600 IU of vitamin D and 500–1000 mg of calcium were administered per day for 8 months in pediatric patients with ALL, from the remission induction phase to the intensification phase. However, despite the supplementation duration, the patients did not reach sufficient vitamin D concentrations, possibly due to the low dose administered [10]. Conversely, in studies carried out by Orgel et al. and Kaste et al., 100,000 IU and 800 IU of vitamin D were administered, respectively, for a longer period (6 months and 2 years), and the patients managed to reach sufficient concentrations [9,11]. However, in our study, we administered a dose of 4000 IU for 6 weeks, and the patients managed to reach these values in a shorter time than that reported by these authors.

Studies have suggested that LCPUFAs-ω3 supplementation acts on the metabolism of vitamin D, increasing its concentration. Partan et al. conducted a study in patients with systemic lupus erythematosus in which they administered 500 uL of seluang fish oil (n = 16) or a placebo (n = 16) for 90 days. The authors reported a significant increase in serum 25(OH)D concentrations in the intervention group, from 44.8 to 89.8 ng/mL, while there were no significant differences in the placebo group. This increase can probably be attributed to the fact that seluang fish is characterized by its rich omega-3 and vitamin D content [31]. However, Salari et al. administered a supplement with 2700 mg of omega-3 fatty acids to postmenopausal women with osteoporosis for 6 months and included a placebo group. They reported a significant increase in 25(OH)D concentrations at 2 and 6 months of supplementation in both groups; however, this effect was not attributed to omega-3 because both groups showed an increase in 25(OH)D [32]. Likewise, in our study, we did not find any effect of omega-3 on serum 25(OH)D concentrations, as both groups showed an increase at the end of the 6-week supplementation period; however, in the ω-3VDCa group, there was a significant decrease in 25(OH)D concentration when supplementation was discontinued compared to the VDCa group, where there were no changes. The difference observed at 12 weeks could be partly explained by the sample size.

In mammals, the de novo synthesis of PTH is regulated in response to alterations in serum calcium, phosphorus, and vitamin D concentrations [33]. A persistent vitamin D deficiency decreases calcium and phosphorus absorption, activating an acute compensatory mechanism by PTH for the release of calcium through an increase in bone resorption, causing a decrease in BMD and mineralization defects [24], and a decrease in PTH concentrations reflects an increase in vitamin D concentrations. At the end of supplementation, we found that the ω-3VDCa group showed a significant decrease in PTH concentrations, reaching a normal level, and, at 6 weeks, PTH tended to be lower than that in the VDCa group (*p* = 0.056). When supplementation was discontinued, both groups showed an increase in PTH concentrations; however, at 12 weeks, the ω-3VDCa group tended to maintain a lower concentration than the VDCa group (*p* = 0.070). This result is consistent with that reported by Hutchins-Wiese et al. In their study, they evaluated the effect of supplementation with 800 IU of vitamin D, 1000 mg of calcium, and 4 g of EPA + DHA (2520 mg of EPA and 1680 mg of DHA) in postmenopausal women who survived breast cancer (n = 17) and a control group that received the same doses of vitamin D and calcium for 3 months (n = 17). They also observed a significant decrease in PTH concentrations at the end of supplementation compared to those at baseline in the group that received EPA + DHA [34]. Even when they administered a higher dose of omega-3 and a lower dose of vitamin D, the effects that they observed were the same as those that we observed regarding the PTH concentrations at 6 weeks (1.5 months), with these effects maintained for another 6 weeks without supplementation; furthermore, our follow-up time was the same as that reported by Hutchins-Wiese.

Bone remodeling is a continuous process involving both bone resorption and formation [35], during which markers of bone resorption (products of type I collagen degradation) and formation (osteoblastic enzymes) are produced [36]. Prostaglandin E2 and proinflammatory cytokines, such as TNF-α, IL-1, and IL-6, induce bone resorption by activating osteoclasts and osteoclastogenesis [37]. In a previous study conducted by our team, we demonstrated a high prevalence of vitamin D deficiency at diagnosis in this population, combined with alterations in bone metabolism, such as an increase in RANKL and a decrease in OPG concentrations, which indicates that these patients experience increased bone resorption [5]. In the search for a nutritional intervention that can influence bone metabolism, the use of nutritional supplements such as vitamin D, calcium, and, more recently, LCPUFAs-ω3 [35], has been suggested. Due to their anti-inflammatory properties, LCPUFAs-ω3 have been shown to inhibit osteoclastogenesis, increase osteoblastogenesis and calcium absorption, and decrease proinflammatory cytokines and prostaglandin E2 [13,34,37].

Regarding the bone resorption marker ICTP at 12 weeks after completing supplementation, the ω-3VDCa group presented significantly lower concentrations than the VDCa group (*p* = 0.024). LCPUFAs-ω3 have been shown to have an effect on bone resorption markers, in accordance with what was reported by Hutchins-Wiese et al., who also observed a decrease in ICTP concentrations between the LCPUFAs-ω3 intervention group and the placebo group in postmenopausal women who survived breast cancer [34]. However, there was no follow-up in patients to determine how long after supplementation this bone resorption marker continued to decrease. Similarly, in patients with osteopenia, Vanlint et al. administered supplements of 400 mg of DHA, 1200 mg of calcium, and 1000 IU of vitamin D daily for 12 months, with a placebo group that received corn oil plus the same doses of calcium and vitamin D (36 women and 4 men). The authors reported a decrease in ICTP at 12 months of supplementation compared to baseline in the group that received DHA; however, they reported no other differences between the groups [38]. Conversely, our linear regression analysis showed a positive effect of DHA on bone metabolism, reflected in a decrease in ICTP concentrations at 12 weeks, which could be explained by the fact that DHA-derived resolvin D1 and neuroprotectin D1 inhibit osteoclastogenesis due to their anti-inflammatory properties [13].

We did not observe changes in OC and TRAP-5b concentrations during or after supplementation. Our results are consistent with those of previous studies conducted by Solmaz and Salari [12,32]. Solmaz et al. administered supplementation with 10 mcg of vitamin D and 100 mcg of vitamin K for 6 months (n = 15), alongside a control group that received only standard treatment (n = 14), and they did not observe any changes in TRAP-5b concentrations during follow-up [12]. Furthermore, Salari et al. administered supplementation with fatty acids for 6 months, and they did not find changes in osteocalcin concentrations [32]. To date, intervention studies have suggested that LCPUFAs-ω3 only decrease resorption markers without having any effect on bone formation [32,34,38].

This is the first study to report the effects of LCPUFAs-ω3, vitamin D, and calcium supplementation on bone metabolism in pediatric patients with ALL, as well as the follow-up of a cohort of patients who received supplementation. The main strengths of our study are that it allows us to begin developing guidelines for the timing of supplementation and the monitoring of 25(OH)D nutritional status, as well as for assessing the duration of an intervention’s effects after it is completed. Additionally, in this study, we used the gold standard method (ultra-high-performance liquid chromatography coupled to a mass spectrometer) to quantify 25(OH)D concentrations. In addition to quantifying the concentrations of 25(OH)D, we also estimated its intake during the supplementation period. Nevertheless, this work has limitations, such as a small sample size due to the lack of adherence to supplementation; a lack of measurement of other markers, in addition to those reported, such as ionic calcium, RANK, RANKL, and OPG; and no long-term follow-up of bone densitometry.

## 5. Conclusions

We confirmed that administering the combination of LCPUFAs-ω3 and 4000 IU of vitamin D for 6 weeks resulted in sufficient concentrations, which were maintained for 6 weeks after the intervention in the VDCa group. This combination had a positive effect on bone health, as indicated by serum ICTP concentrations, without affecting serum 25(OH)D levels. The vitamin D deficiency present in these patients is a trigger for long-term bone health complications, such as failure to reach peak bone mass, osteoporosis, and fractures; thus, it is necessary to establish supplementation strategies and to question the temporality of using a vitamin D intervention to improve the quality of life of childhood ALL survivors.

## Figures and Tables

**Table 1:** Demographic and clinical characteristics for all randomized and study-completing children by protocol.

	Baseline (Randomized)		6 Weeks		12 Weeks	
**Variables**	**ω** **-3VDCa** **(n = 19)**	**VDCa** **(n = 17)**	**ω** **-3VDCa** **(n = 12)**	**VDCa** **(n = 7)**	**ω** **-3VDCa** **(n = 12)**	**VDCa** **(n = 7)**
**Sex**						
Female n (%)	10 (52.6)	6 (35.3)	5 (41.7)	3 (42.9)	5 (41.7)	3 (42.9)
Age at study entry (years)	9.0 (6.6,13.8)	10.0 (7.1,16.0)	7.0 (5.7,9.0)	10 (7.1,15.1)	7 (5.7, 9.0)	10 (7.1, 15.1)
**Nutritional status**						
Weight (kg)	30.7 (21.6,48.6)	42.9 (23.4,51.9)	23.7 (20.3,29.5)	46.5 (25.4,50.2)	24.7 (20.9,29.3)	47.6 (25.0,50.3)
Height (cm)	127.1 (116,151.9)	139.6 (123.1,162.6)	119.3 (114.5,135.4)	142.6 (127.5,162)	119.8 (116.2,135.9)	143.6 (128.5,166.8)
Eutrophic (BMI pc >5 to pc <85)	14 (73.7)	9 (52.9)	10 (83.3)	6 (85.7)	9 (81.8)	6 (85.7)
Overweight (BMI pc >85 to pc <95)	4 (21.1)	4 (23.5)	-	-	-	-
Obesity (BMI pc >95	1 (5.3)	4 (23.5)	1 (8.3)	1 (14.3)	1 (9.1)	1 (14.3)
Fat (%)	28.7 (24.5,35.2)	25.1 (20.5,37.1)	26.2 (23.3,28.6)	22.8 (18.0,29.1)	26.0 (21.8,28.6)	20.9 (17.3,23.1)
**Risk stratification**						
Habitual/intermediate risk, n (%)	6 (31.6)	8 (47.1)	5 (41.7)	3 (42.9)	5 (41.7)	3 (42.9)
High risk, n (%)	13 (68.4)	9 (52.9)	7 (58.3)	4 (57.1)	7 (58.3)	4 (57.1)

Data are presented as mean ± standard deviation (SD), median (25th–75th percentile), or as a number and percentage. BMI: body mass index. ω-3VDCa group: omega-3 long-chain polyunsaturated fatty acids, vitamin D, and calcium; VDCa group: vitamin D and calcium. Data was analyzed with Student’s *t* test for independent samples or the Mann–Whitney U test; Chi square or Fisher’s exact test was used according to the variable’s distribution.

**Table 2:** Bone mineral density according to nutritional status of vitamin D at baseline.

BMD L1–L4 Z-Score	Total Sample (n = 36)	Deficiency/Insufficiency (n = 33)	Sufficiency (n = 3)
>−1 SD Normal, n (%)	21 (58)	21 (64)	-
−1 SD and −2 SD Osteopenia, n (%)	11 (31)	10 (30)	1 (33)
≤2 SD Osteoporosis, n (%)	4 (11)	2 (6)	2 (67)

Data are presented as numbers and percentages. SD: standard deviation; BMD: bone mineral density; L1–L4: lumbar vertebrae 1 to 4; deficiency < 19.9 ng/mL; insufficiency 20–29 ng/mL; sufficiency > 30 ng/mL.

**Table 3:** Multiple linear regression model for predictors of ICTP concentrations at 12 weeks.

ICTP		
	**β**	***p***
DHA (%)	−0.630	0.012
25(OH)D (ng/mL)	−0.339	0.146
Model	R^2^ = 0.341	0.036

ICTP: *C*-terminal telopeptide of type I collagen; DHA: docosahexaenoic acid; 25(OH)D: 25-hydroxyvitamin D.

## Data Availability

The data are not publicly available due to, privacy, legal or ethical reasons. The data presented in this study are available upon request from the corresponding author.

## Abbreviations

The following abbreviations are used in this manuscript:

ALL	Acute lymphoblastic leukemia
LCPUFAs-ω3	Omega-3 long-chain polyunsaturated fatty acids
ω3VDCa	Omega-3 long-chain polyunsaturated fatty acids, vitamin D, and calcium group
VDCa	Vitamin D and calcium group
25(OH)D	25-hydroxyvitamin D
Ca	Calcium
PTH	Parathormone
ICTPs	Human cross-linked C-terminal telopeptides of type I collagen
TRAP-5b	Human tartrate-resistant acid phosphatase 5b
OC	Osteocalcin
MSCs	Mesenchymal stem cells
RANKL	Receptor Activator of Nuclear Factor κB Ligand
OPG	Osteoprotegerin
DHA	Docosahexaenoic acid
EPA	Eicosapentaenoic acid
M-CSF	Macrophage colony-stimulating factor
MITF	Microphthalmia-associated transcription factor
RANK	Receptor Activator of Nuclear Factor κB
SR	Standard risk
IR	Intermediate risk
HR	High risk
BMI	Body mass index
DXA	Dual-energy X-ray absorptiometry
BMD	Bone mineral density
SD	Standard deviation
